# Supplementary material for: Stable de novo protein design via joint conformational landscape and sequence optimization
Source: Nat Commun. 2025 Dec 24;17:8. doi: 10.1038/s41467-025-66526-w (PMC12764529; doi:10.1038/s41467-025-66526-w)
Supplement: Supplementary file 1 — Supplementary Information [file 41467_2025_66526_MOESM1_ESM.pdf]

Supplementary Information

Stable De Novo Protein Design via Joint Conformational Landscape and Sequence Optimization

Yehlin Cho[1], Justas Dauparas[2], Kotaro Tsuboyama[3,4], Gabriel J. Rocklin[3,*], Sergey Ovchinnikov[1,*]

[1]Massachusetts Institute of Technology, 77 Massachusetts Ave, Cambridge, MA 02139, USA
[2]Institute for Protein Design, University of Washington, Seattle, WA, USA
[3]Department of Pharmacology & Center for Synthetic Biology, Northwestern University Feinberg School of Medicine, Chicago, IL, USA
[4]Institute of Industrial Science, The University of Tokyo, Tokyo, Japan

[*]Correspondence: grocklin@gmail.com, so3@mit.edu

Supplementary Figures

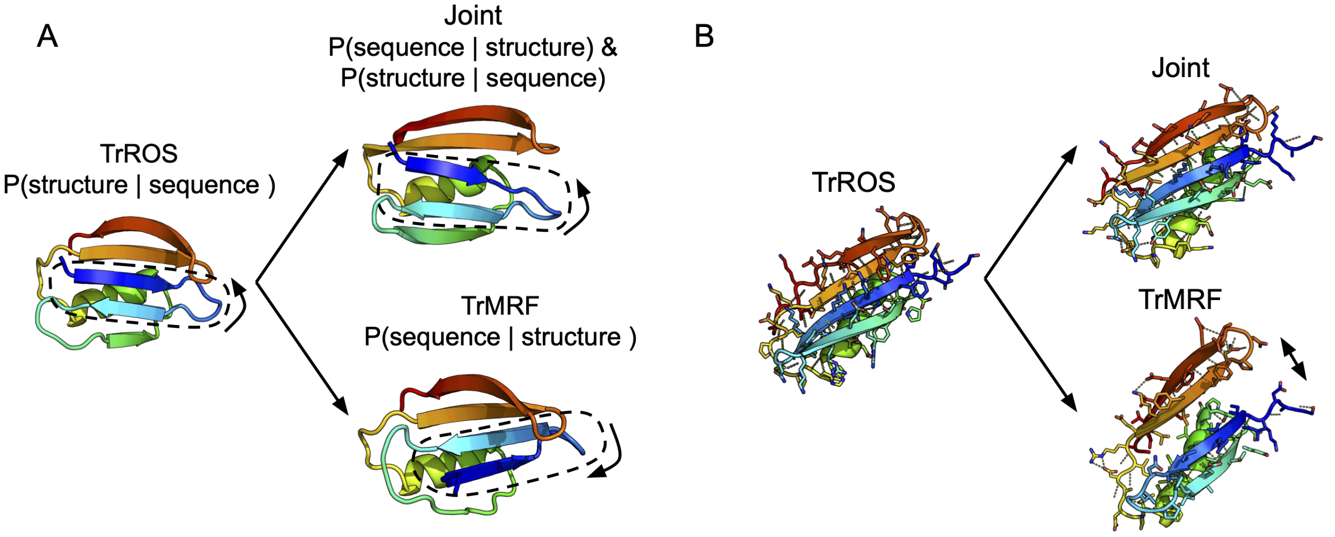

Supplementary Figure 1. Failure cases of the $P(\text{sequence}|\text{structure})$ model performing inverse folding. Structures redesigned using the joint model and TrMRF model are compared, given the TrROS structure as the scaffold. (A) Swapping of β-sheet strands in the structure designed by TrMRF. (B) Disruption of backbone interactions and distances in the structure designed by TrMRF.

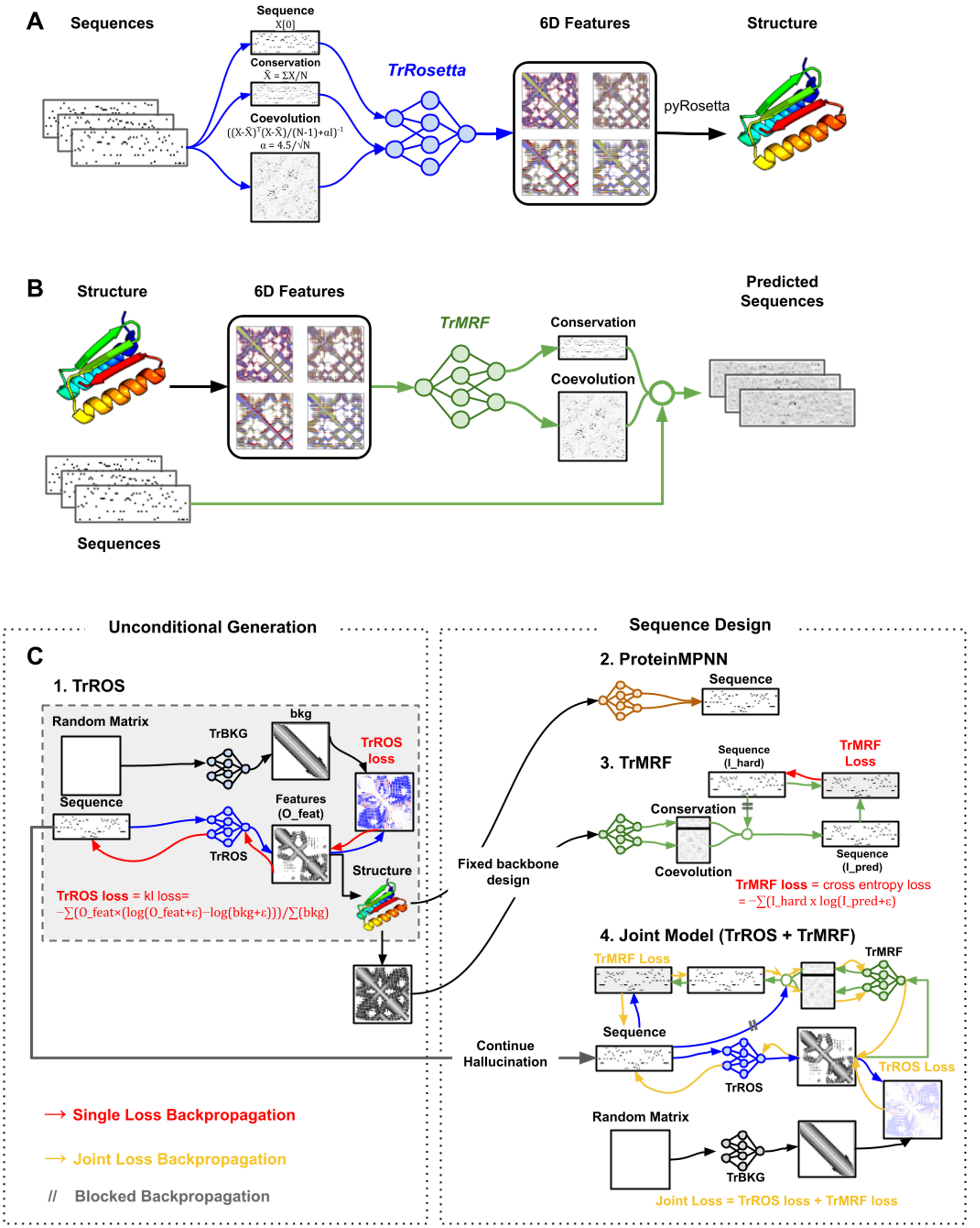

Supplementary Figure 2. De novo Protein Sequence and Structure Generation: (A) Model architecture of TrROS, (B) Model architecture of TrMRF, and (C) Overview of end-to-end generation of sequences using four different models. I_{true} and I_{pred} are the sequence logits given and predicted, O_{feat} represents the predicted 6D features, and bkg refers to the background 6D features.

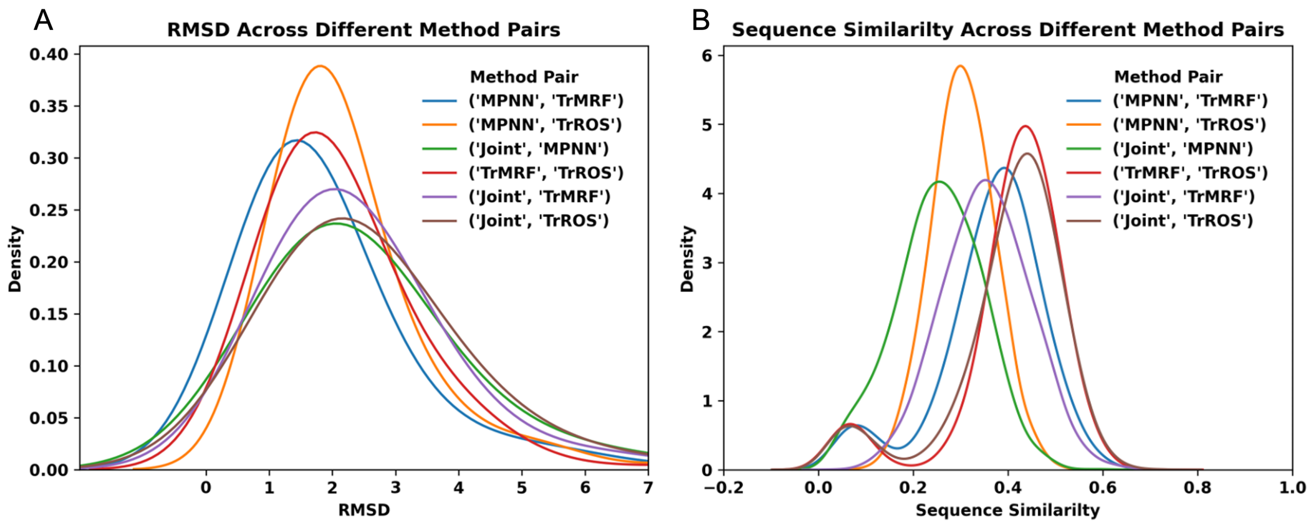

Supplementary Figure 3. (A) Structure similarity measured by RMSD across different pairs of methods for structures filtered by AlphaFold pLDDT scores higher than 70 and inter-PAE higher than 15 (a total of 5,167 structures). (B) Sequence similarity across different pairs of methods (a total of 20,668 sequences)

A

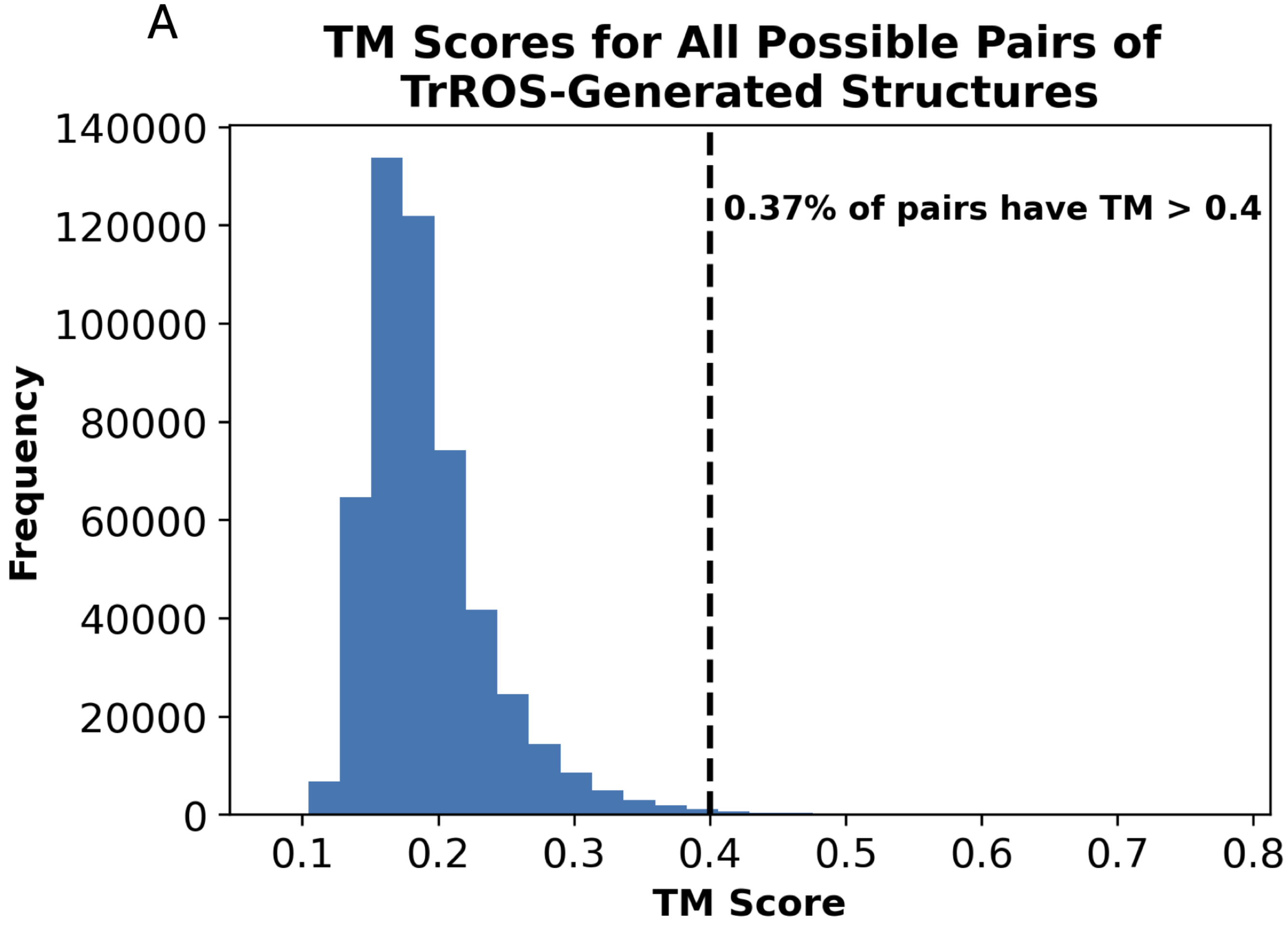

Supplementary Figure 4. (A) TM scores between TrROS-generated structures: Generally, if the TM score is less than 0.4, the two proteins are not considered to be in the same fold. Here, 0.37% of pairs have a TM score greater than 0.4, so most of them are different.

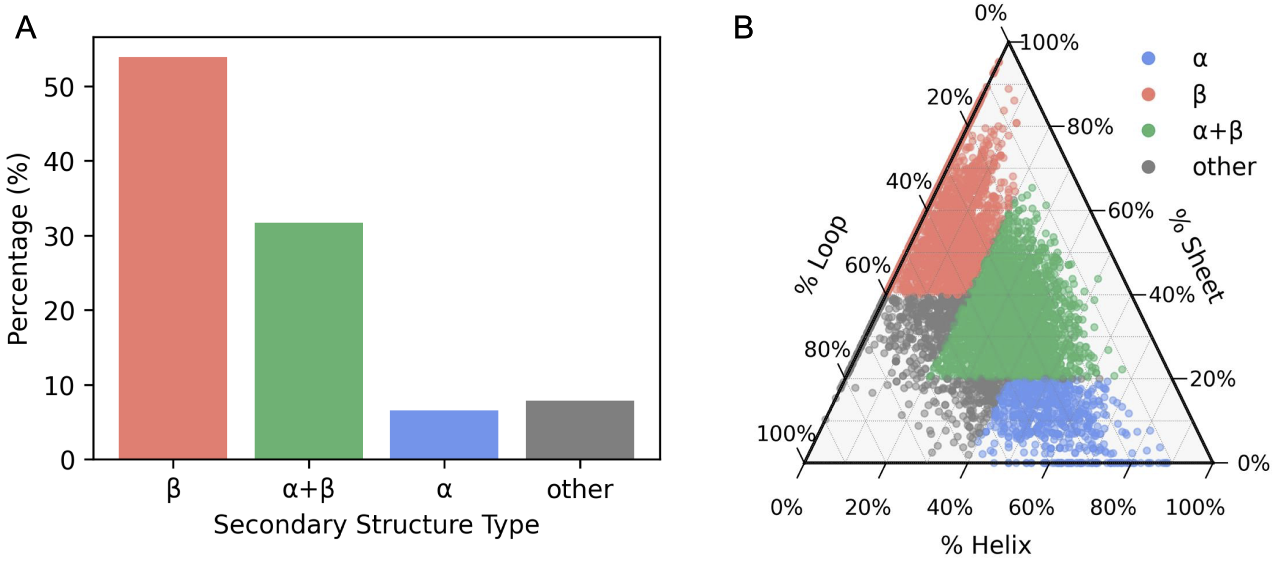

Supplementary Figure 5. (A) Overall percentage of secondary structure classes in the generated structures. Secondary structure classes are defined as follows: if helix content is over 40% and sheet content is under 20%, the structure is classified as α; if sheet content is over 40% and helix content is under 20%, it is classified as β; if both helix and sheet contents exceed 20%, it is classified as $\alpha\beta$; otherwise, it is classified as other. (B) Scatter ternary plot showing the composition of helix, sheet, and loop as calculated by DSSP. Each scatter point represents a single structure.

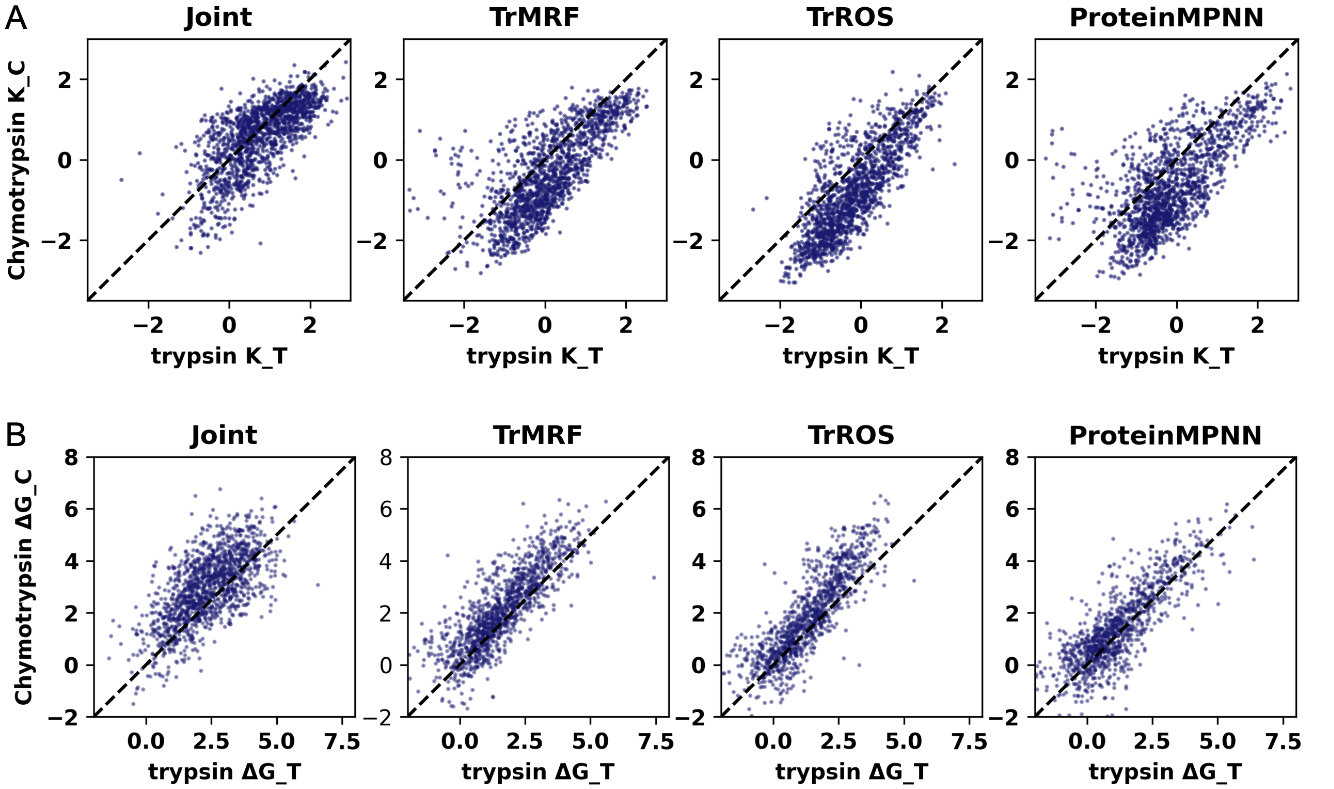

Supplementary Figure 6. Comparisons of (A) resistance to trypsin (k_T) vs. chymotrypsin (k_C) and (B) folding stability to trypsin (ΔG_T) vs. chymotrypsin (ΔG_C).

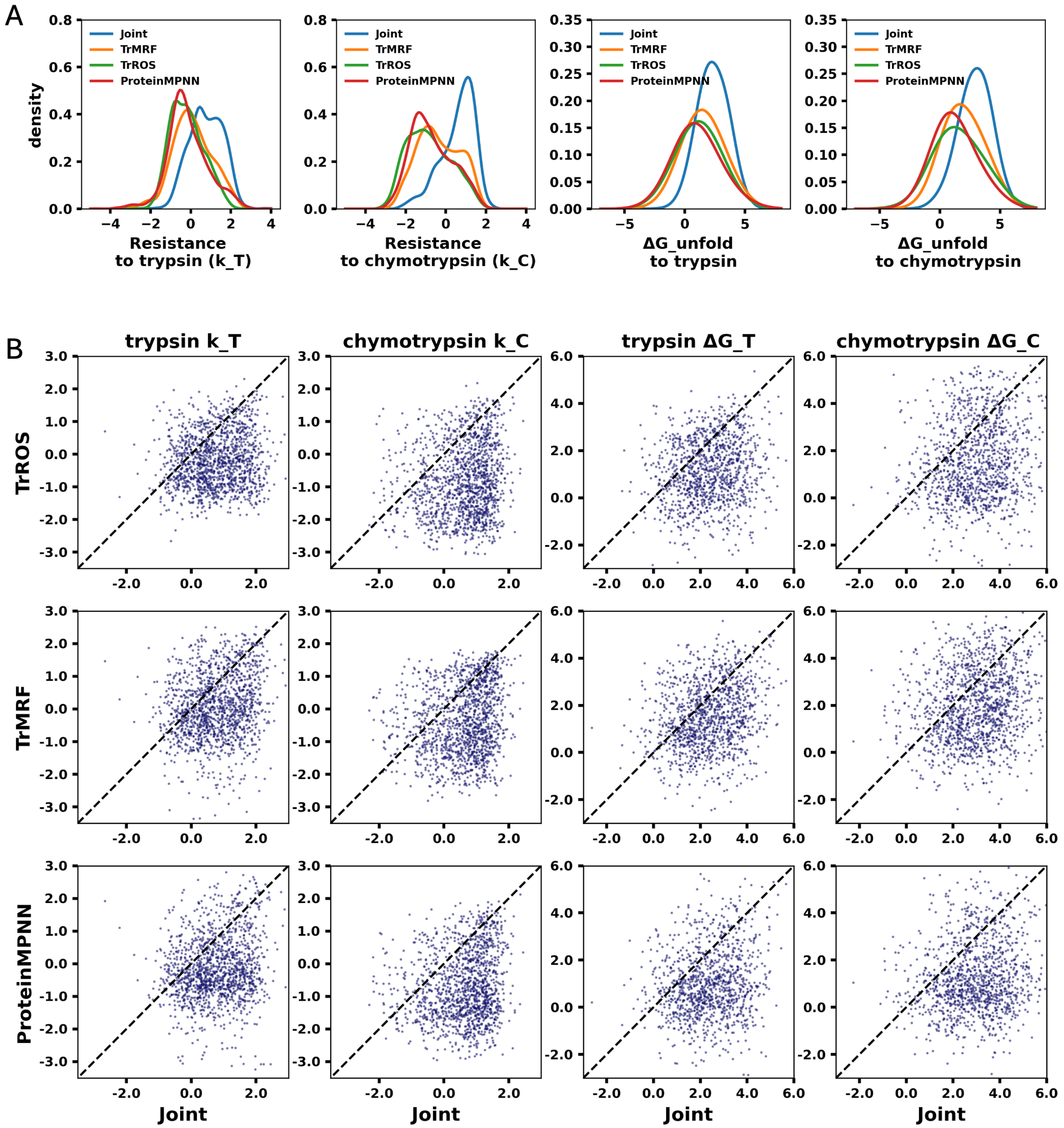

Supplementary Figure 7. Experimental metrics comparisons of folding stability between different models. (A) resistance to trypsin (k_T), chymotrypsin (k_C), folding stability to trypsin (ΔG_T), and chymotrypsin (ΔG_C). (B) Folding stability comparisons between single models and the joint model0 using the same metrics as in (A).

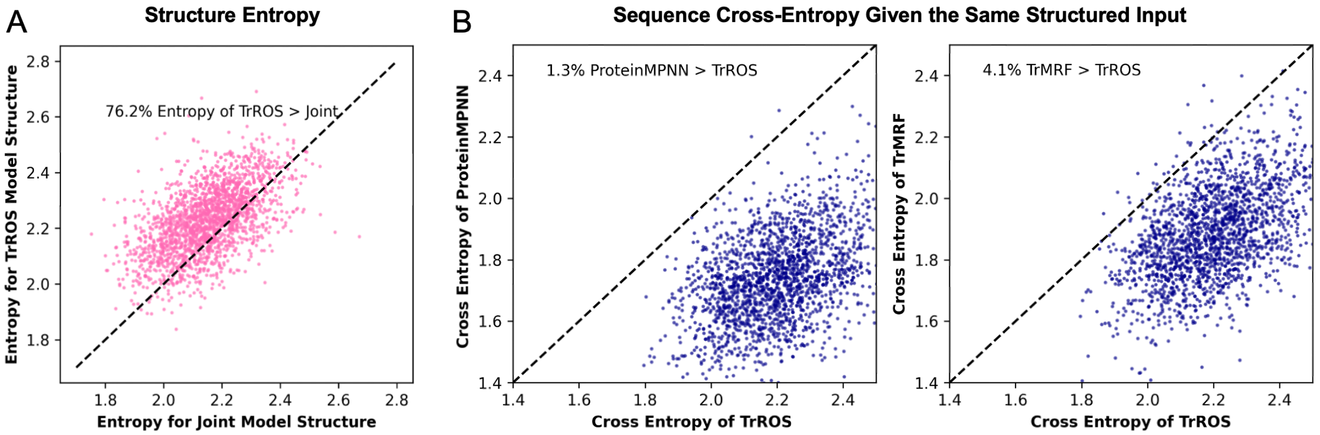

Supplementary Figure 8. Approximation of Structure Probability and Sequence-Structure Compatibility Using ProteinMPNN Confidence Score. (A) Comparing the structural entropy of the TrROS output structure with the Joint model output structure to approximate $P(\text{structure})$, in order to compare the suboptimal structure and the adjusted structure produced by the Joint model. (B) Evaluating sequence cross-entropy, given the same input structure, by comparing sequences from $P(\text{sequence} \mid \text{structure})$ with $P(\text{structure} \mid \text{sequence})$, to assess how well the sequence aligns with the given structure. On the left, this comparison is made between TrROS and ProteinMPNN, while on the right, it is made between TrROS and TrMRF. High entropy and cross-entropy indicate higher uncertainty in the model, whereas low entropy and cross-entropy suggest greater confidence and better alignment between sequence and structure.

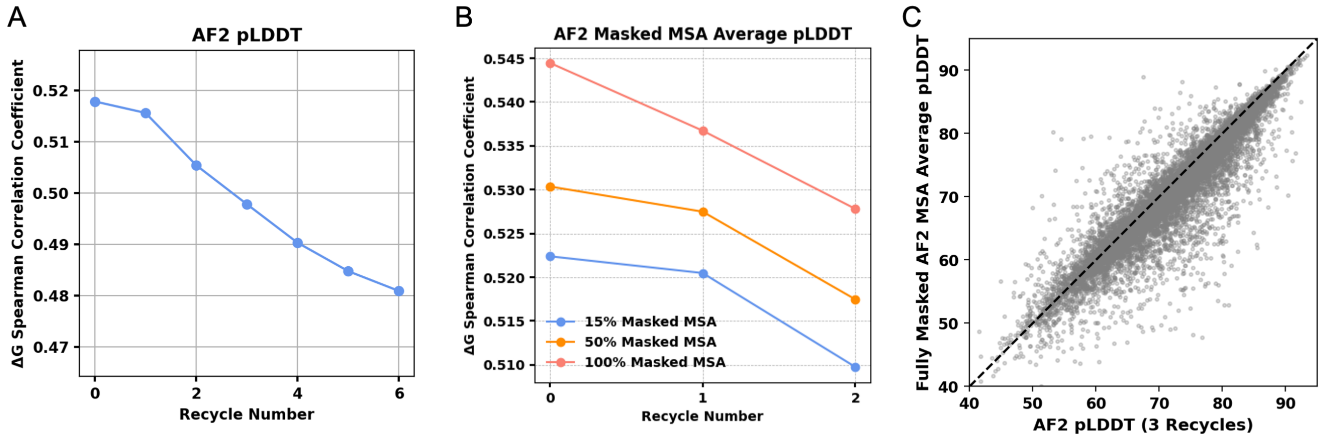

Supplementary Figure 9. Correlation between AF2 pLDDT and experimental folding stability under different conditions: (A) varying recycle numbers, (B) varying percentages of MSA masking, and (C) comparison of AF2 pLDDT predictions with 3 recycles versus fully masked MSA input without recycling.

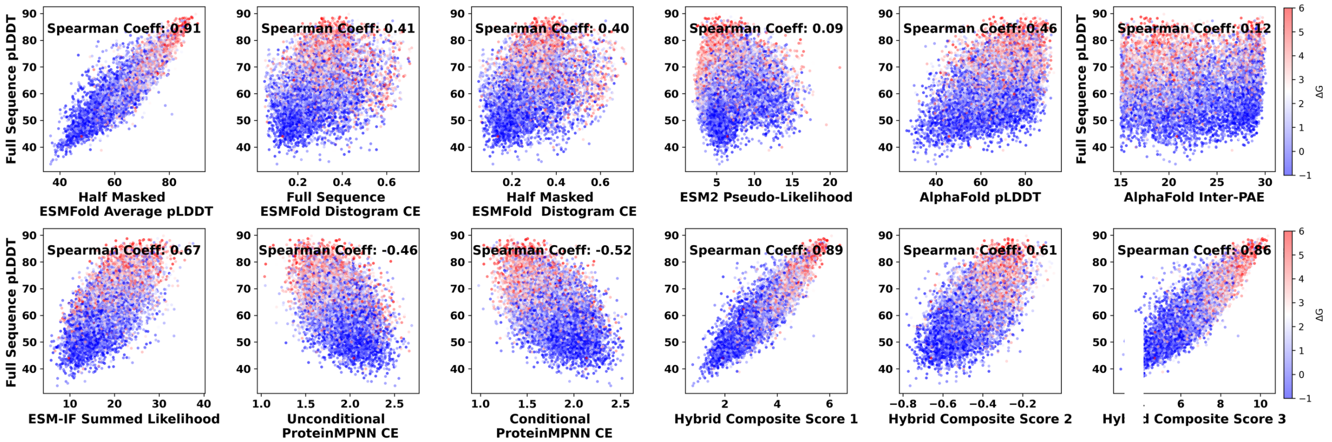

Supplementary Figure 10. Comparison of the models' folding stability predictions in a zero-shot setting with the full-sequence-based ESMFold pLDDT score. The plotted data points are colored by ΔG_{unfold} values, with red indicating positive values and blue indicating negative values.

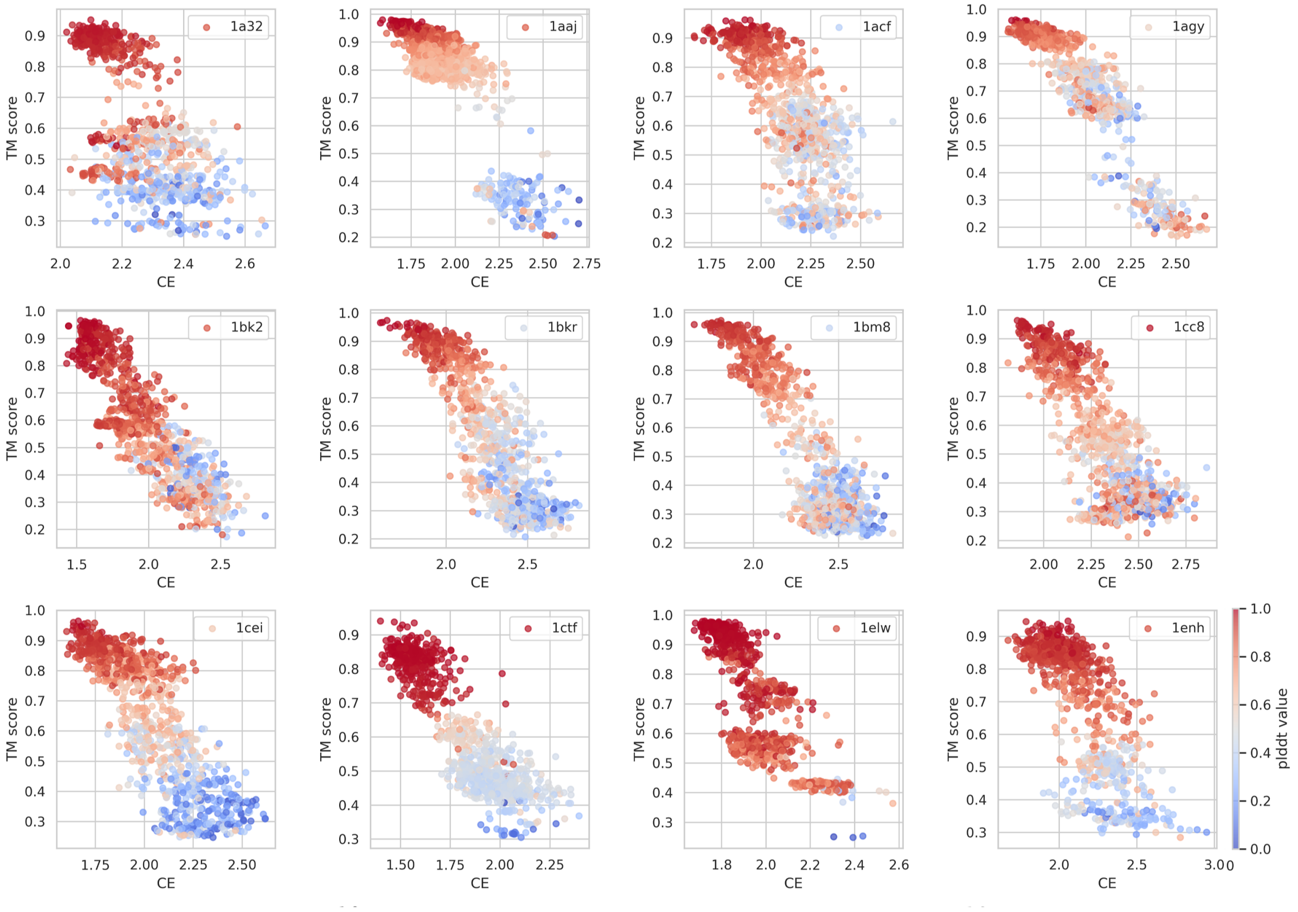

Supplementary Figure 11. Scatter plot of TM score and ProteinMPNN cross-entropy (CE), colored by Alphafold2 pLDDT.

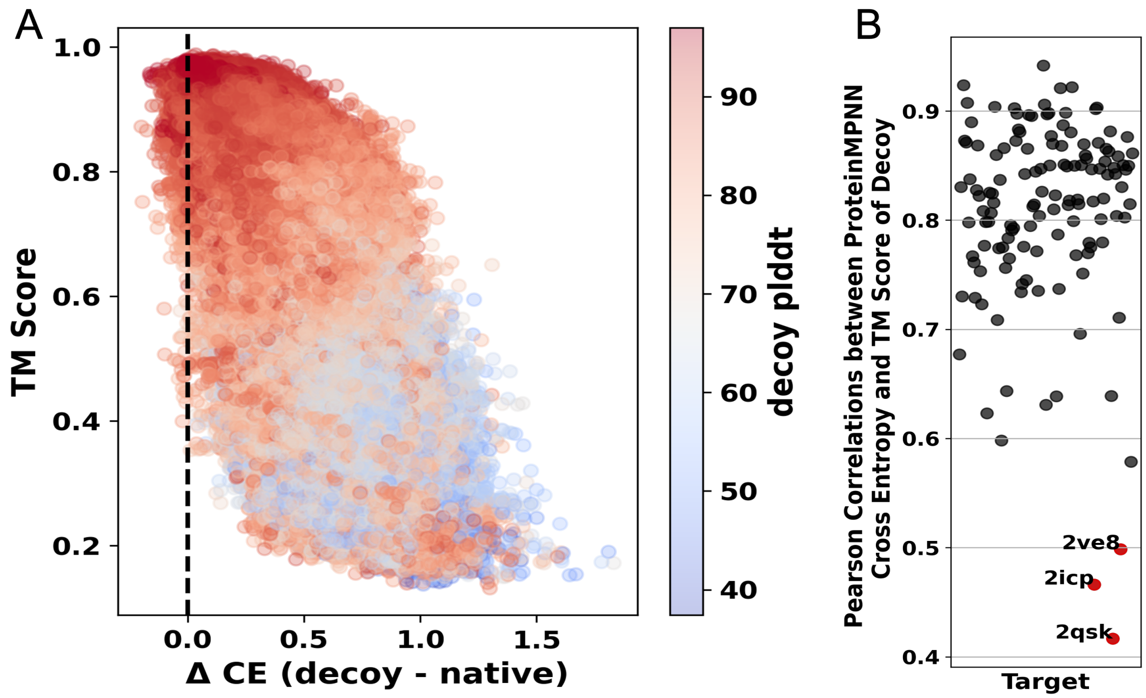

Supplementary Figure 12. Correlation of TM score and CE for(A) total decoys (B) each individual target decoy.

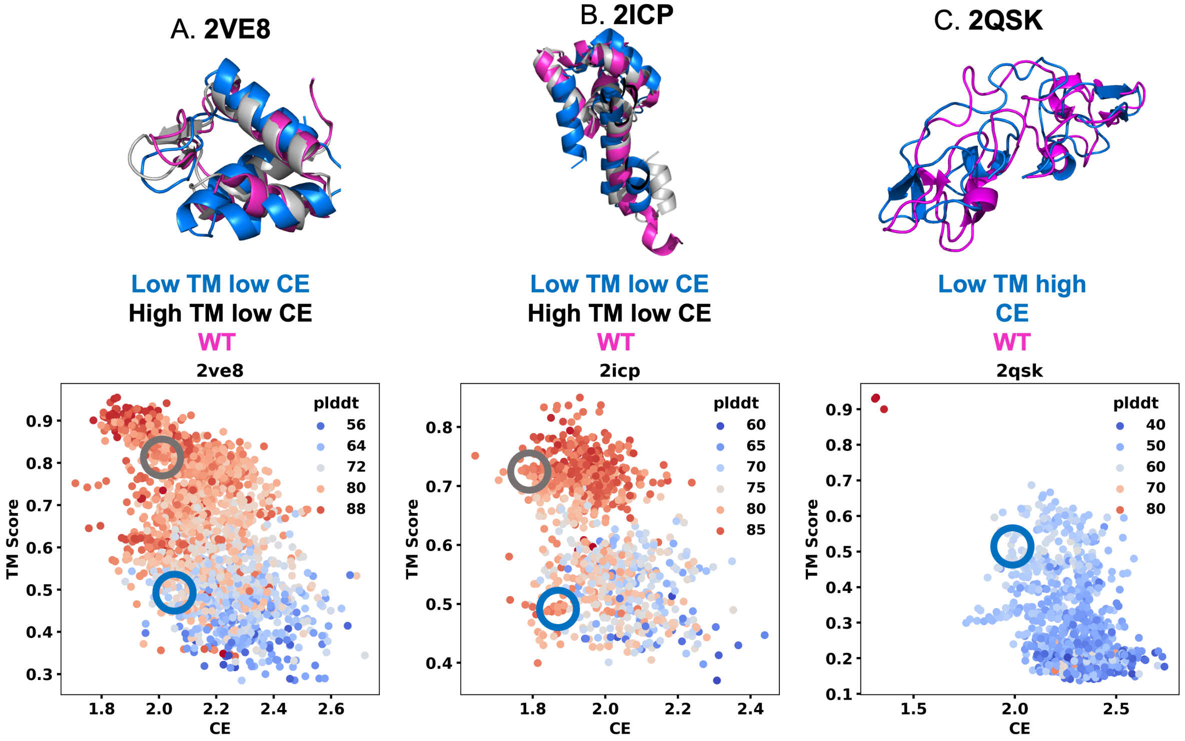

Supplementary Figure 13. Three representative structures show less correlation between the TM score and ProteinPNN cross entropy (CE): A. 2VE8, B. 2ICP, C. 2QSK structures. The top figures show overlapped structures of the wild type and decoy structure, indicating a high TM score and a low TM score with the wild type.

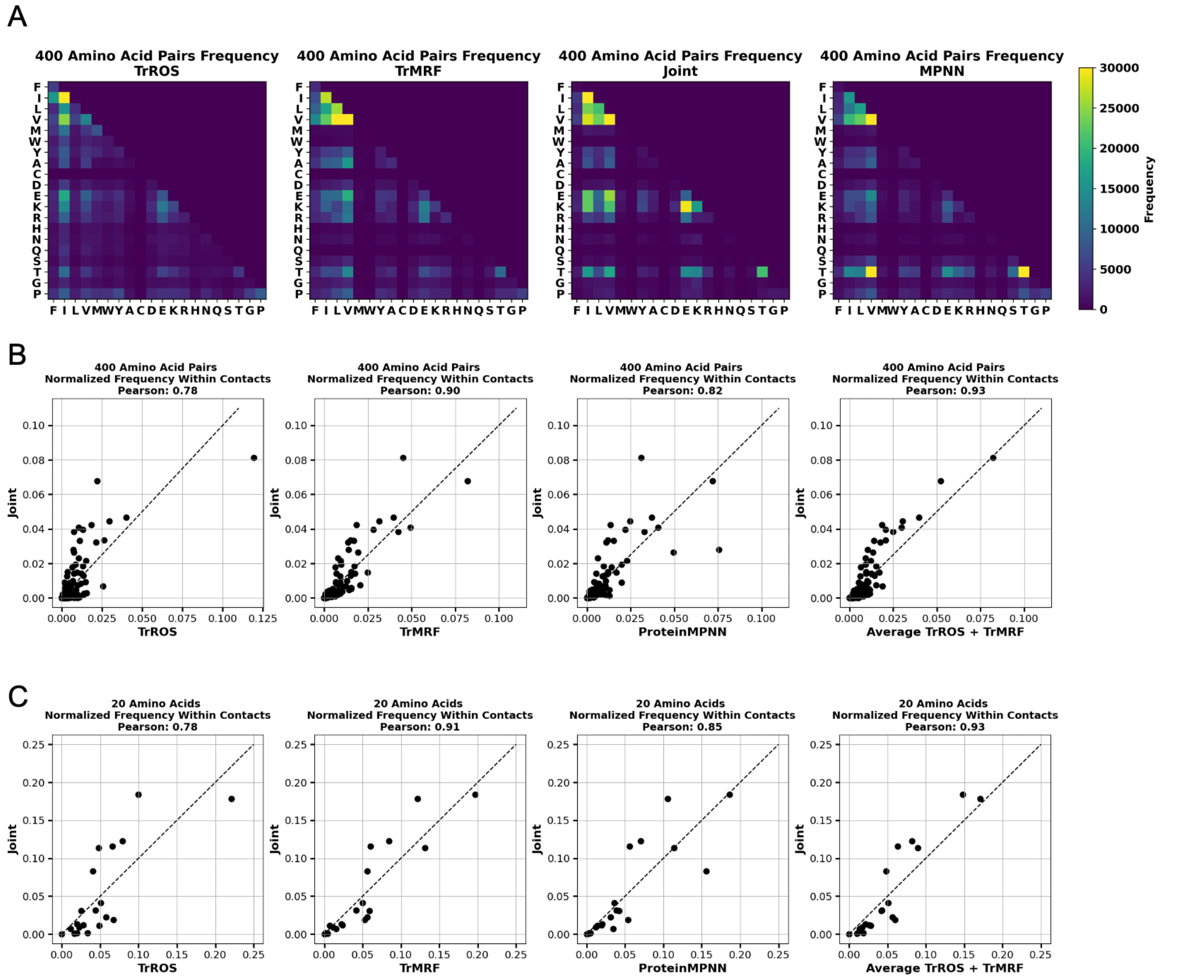

Supplementary Figure 14. Amino Acid Frequency in Contacts. (A) Frequency of 20x20 amino acid pairs in contacts across sequences generated from four different models. Scatter plots compare (B) the normalized frequency of 400 amino acid pairs and (C) the frequency of 20 single amino acids between the single model and the joint model.

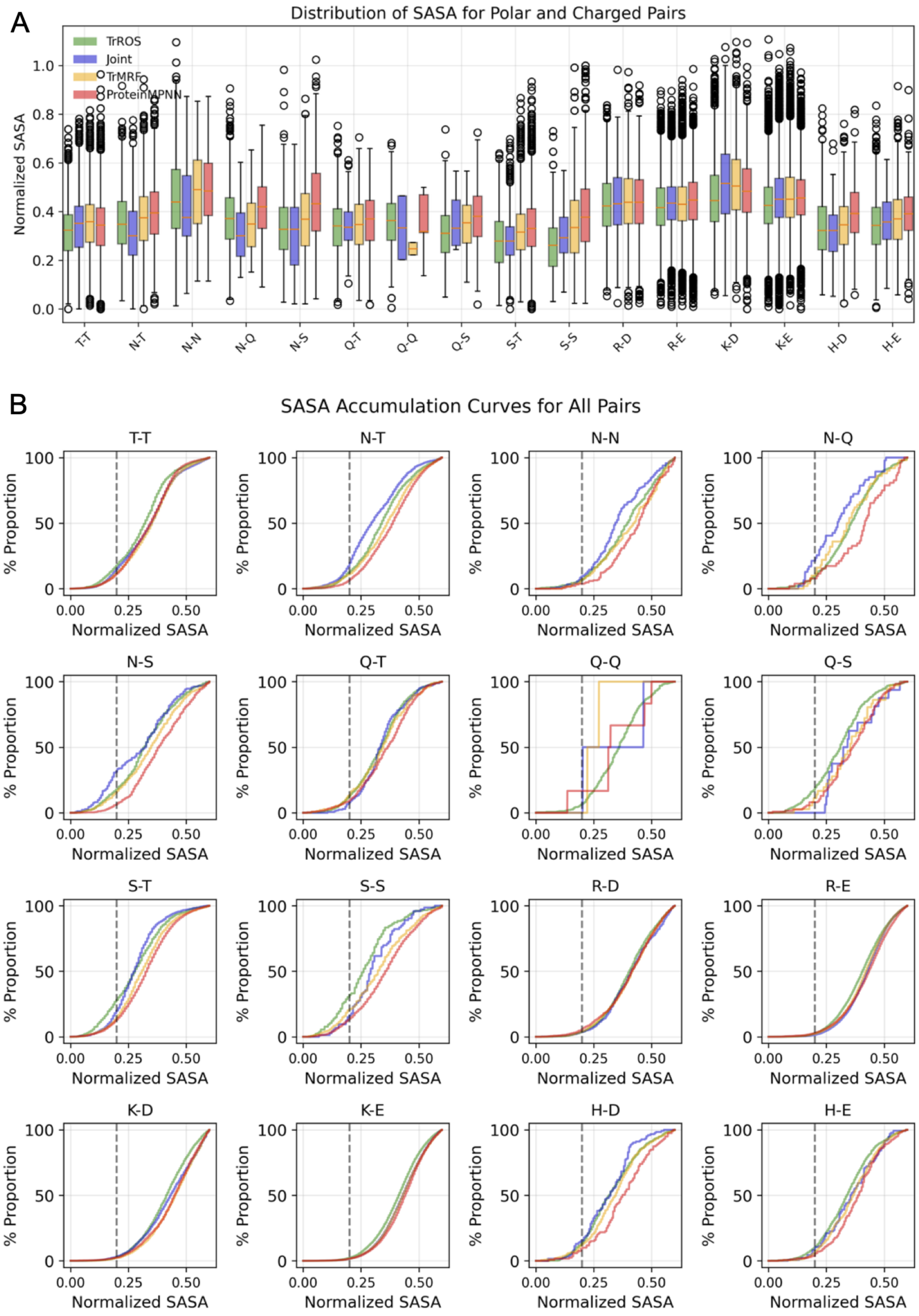

Supplementary Figure 15. Analysis of solvent accessibility in residue pairs across protein design models. (A) Distribution of solvent accessible surface area (SASA) for polar and charged residue pairs in designs generated by all four models. (B) Cumulative distribution of normalized SASA per residue pair, showing the fraction of pairs with normalized SASA below indicated threshold values. For certain amino acid pairs, such as asparagine pairing with serine, glutamine, and threonine, the joint model shows a higher proportion of buried pairs (normalized SASA < 0.2). ProteinMPNN exhibits higher normalized SASA across most residue pairs, indicating greater surface exposure compared to other models. SASA was calculated using the Shrake-Rupley method, with normalization by dividing each residue's SASA by the maximum SASA value reported for that residue type.

Supplementary Method

A. cDNA Display-based Folding Stability Measurement

1. Analyzing protein folding stability via proteolysis in the cDNA display framework

In the case of many small, single-domain proteins, folding can be simplified as a two-state system. This system involves two distinct populations of proteins—those in a folded state and those in an unfolded state—separated by a free-energy barrier [42]. Folding stability, in this context, is measurable as the difference in free energy levels between the folded and unfolded states, denoted as the folding free energy (ΔG_{Fold}). This can be experimentally measured using K50, where the unfolded state is denoted as $K50_{\mathrm{U}}$ and the folded state as $K50_{\mathrm{F}}$. Tsuboyama, Kotaro, et al. introduce a predictive tool for assessing the folding stability of unfolded proteins ($K50_{\mathrm{U}}$) under protease conditions, called No U-Turn Sampler (NUTS), using a dataset comprising approximately 64,238 protein scramble proteolysis samples [13]. The model can distinguish stable sequences from unstable ones through protease treatment. Furthermore, a robust correlation emerges between ΔG values obtained from cDNA display proteolysis and known folding ΔG values [13]. To validate the folding stability of our designed proteins, we conduct a DNA display-based proteolysis experiment using two different proteases: trypsin, which selectively acts on basic amino acids, and chymotrypsin, which targets amino acids with aromatic properties.

2. Experiment Details of high-throughput display-based cDNA proteolysis

To measure the folding stability of proteins, we used proteolysis reactions. We assumed single-turnover kinetics [43] with a substantial excess of enzyme compared to the substrate. Equation (4) describes a proteolysis process in which the protease (E) and the folded protein (F) can form an enzyme-substrate complex (FE), leading to proteolysis and degradation into a product (P). Furthermore, the enzyme can proteolyze the unfolding protein (U) to form an unfolded enzyme-substrate complex (UE), resulting in degradation and the production of a decorated protein (P). There is an equilibrium between the folded and unfolded states of the protein, and the free energy between the two states represents the folding stability ΔG in equation (5). The bi-directional arrows indicate the reversible process.

$$
\begin{gathered}
E+F \rightleftharpoons FE \rightarrow E+P \\
E+U \rightleftharpoons UE \rightarrow E+P
\end{gathered}
\tag{4}
$$

$$
\Delta G, Unfold = -RT \ln\left(\frac{[U]}{[F]}\right) \tag{5}
$$

If $\Delta G_{,\mathrm{Unfold}}$ is positive, it indicates that the folded state is more stable than the unfolded state. The calculation of $\Delta G_{,\mathrm{Unfold}}$ relies on the concentrations of unfolded and folded proteins, specifically using the ratio $[U]/[F]$. This ratio can be obtained the measured sequence-specific K50 (the protease concentration at which the cleavage rate is half of the maximum rate, kmax), unfolded state (K50,U), and folded state (K50, F), as shown in equation (6). Here, we set K50, F to be same for all sequences, because we assume that folded state cleavage occurs only within the constant regions of the construct such as the N-terminal PA tag which is used to pull down the proteins [13].

$$
\begin{aligned}
K_{50} \approx K_d = \frac{[E][S]}{[ES]} &= \frac{([U]+[F])[E]}{([UE]+[FE])} \\
&\approx \frac{[U]+[F]}{[U]/K_{50,u}+[F]/K_{50,f}}
\end{aligned}
\tag{6}
$$

$$
\frac{[U]}{[F]} \approx \frac{1/K_{50}-1/K_{50,f}}{1/K_{50,u}-1/K_{50}} \tag{7}
$$

Proteolysis reactions are conducted under two conditions: with trypsin and with chymotrypsin. For each group, experiments are performed across 11 different protease concentrations, arranged in a threefold dilution series, with one condition without protease to determine the point at which the cleavage rate is half of Kmax (K50)[13]. Bayesian inference is employed to derive K50 and ΔG values for all sequences within our library. This analysis comprises two main models. The first model, referred to as the 'K50 model,' calculates the K50 values for each sequence based on the count data from sequencing. The second model, named the 'unfolded state model', anticipates the unfolded state K50 value (K50,U) for each sequence by considering its actual sequence [13]. As shown in Figure 6, there is a clear linear correlation between ΔG values of trypsin and chymotrypsin.

B. Details of Folding Stability Predicting Models

We plot one-to-one comparison scatter plots between individual prediction scores (Figure 10). The x-axis includes the half-masked ESMFold pLDDT, full and half-masked ESMFold Distogram cross-entropy, ESM2 pseudo-likelihood, AlphaFold2 pLDDT, ESM-IF summed likelihood, Unconditional ProteinMPNN CE, Conditional ProteinMPNN CE, and hybrid scores 1, 2, and 3. The y-axis is fixed to Full sequence ESMFold pLDDT.

C. Spearman comparisons between TM (template modeling score) and CE (cross entropy) of ProteinMPNN

We hypothesize that ProteinMPNN, which is trained on structure space from the PDB dataset, can evaluate the difference between proteins in their lowest energy conformation and decoys created using relaxation or backrub methods. We can approximate how well the ground truth sequence matches the given structure by calculating the cross-entropy loss between the output logits and the ground truth sequence. To test this hypothesis, we prepared 134 targets. For each target, we sampled 1,000 Rosetta decoy structures [23]. We then provided these sampled structures into the ProteinMPNN model, predicted logits, and calculated the categorical cross-entropy loss between these logits and the wild-type sequence.

When we plotted the cross-entropy (CE) and TM scores between decoy and native structures, a clear inverse linear correlation emerged: as cross-entropy decreases, the similarity between the two structures increases. With a total of 134 targets and 1,300 decoy structures per target, we observed a high Pearson correlation, as shown in Figure 11 and Figure 12. Three representative structures showed relatively low correlations, between 0.4 and 0.5. These proteins either have disordered regions, causing sampled decoys from Rosetta to have a low TM score with the wild-type structure, or the disordered regions result in different conformations while sampling the decoys (Figure 13).
