# Supplementary material for: Stable de novo protein design via joint conformational landscape and sequence optimization
Source: Nat Commun. 2025 Dec 24;17:8. doi: 10.1038/s41467-025-66526-w (PMC12764529; doi:10.1038/s41467-025-66526-w)
Supplement: Supplementary file 2 — Description of Additional Supplementary Files [file 41467_2025_66526_MOESM2_ESM.pdf]

Description of Additional Supplementary Files

File Name: Supplementary Data 1
Description: This file contains protein model confidence scores, used as a proxy for stability, for sequences generated by four design models: TrRos, TrMRF, ProteinMPNN, and the Joint model.
